# Ulinastatin ameliorates acute kidney injury following liver transplantation in rats and humans

**DOI:** 10.3892/etm.2014.2088

**Published:** 2014-11-25

**Authors:** XIAOYUN LI, XIANG LI, XINJIN CHI, GANGJIAN LUO, DONGDONG YUAN, GUOLIANG SUN, ZIQING HEI

**Affiliations:** Department of Anesthesiology, Third Affiliated Hospital, Sun Yat-sen University, Guangzhou, Guangdong 510630, P.R. China

**Keywords:** orthotopic liver transplantation, acute kidney injury, ulinastatin

## Abstract

Acute kidney injury (AKI) is a common complication following orthotopic liver transplantation (OLT) that evidently affects prognosis. However, no effective treatment exists for AKI. The aim of the present study was to elucidate whether ulinastatin application during OLT in humans can reduce kidney damage and improve renal function. In addition, the underlying mechanisms of ulinastatin were investigated on a rat autologous OLT (AOLT) model. In total, 60 patients undergoing an OLT were randomly selected to receive ulinastatin (U group; n=30) or saline (C group; n=30) during the OLT surgery. The patient demographics, AKI incidence rate, recovery indicators and renal injury indexes were measured during the perioperative period. In addition to the clinical trials, 40 rats were subjected to an AOLT and were divided into the control (C-R), sham-operation and ulinastatin treatment groups. Pathological renal damage, biomarkers of inflammation and oxidative stress were measured to investigate the effects and possible mechanisms of ulinastatin on AKI. In the clinical trials, ulinastatin application was shown to attenuate the incidence of AKI following OLT (P<0.05) and reduce the serum levels of cystatin C and urinary β_2_ microglobulin within 24 h of the OLT (P<0.05). Furthermore, ulinastatin was found to significantly improve the recovery of patients by reducing the time spent in the intensive care unit (P<0.01 vs. C group), the ventilation time and the hemodialysis rates (P<0.05 vs. C group). In the rat AOLT model, ulinastatin application was also shown to relieve renal pathological damage by reducing the serum cystatin C and creatinine levels. Notably, the levels of tumor necrosis factor-α, interleukin-6, hydrogen peroxide and reactive oxygen species were evidently reduced, while the level of superoxide dismutase was increased in the ulinastatin groups (P<0.05, vs. C-R group). In conclusion, ulinastatin application was demonstrated to protect against AKI following OLT by inhibiting inflammation and oxidation.

## Introduction

Acute kidney injury (AKI) is a common and significant complication (incidence, 17–95%) affecting the prognosis of patients undergoing orthotopic liver transplantation (OLT) (1,2). The deterioration of renal function is due to various etiologies, including preoperative hepatorenal pathological damage, multiple injury factors in the perioperative period and nephrotoxic therapies following surgery (3). Although, various treatments targeting AKI have been provided to patients following OLT, the results remain unsatisfactory. Thus, developing an effective method for AKI therapy following OLT is essential.

Ulinastatin, a glycoprotein derived from human urine, has a molecular weight of 67 kDa and functions as an inhibitor of a number of proteases, including trypsin, chymotrypsin, elastase and various pancreatic enzymes (4). Several clinical studies and animal experiments have reported that the application of ulinastatin can play an important role in the protection against septic shock, intensive pancreatitis, ischemia-reperfusion organ injury and multi-organ dysfunction; it is believed that this protection is associated with the anti-inflammatory effects of ulinastatin (5–8). Although ulinastatin has been widely used in clinical practice, studies investigating the effect of ulinastatin on the renal protection of patients during the OLT perioperative period are limited, and the underlying mechanisms through which ulinastatin reduces pathological damage remain unclear. Therefore, the present study investigated the influence of ulinastatin on AKI, which is the most significant complication of OLT.

Hemodynamic fluctuation during OLT is extremely intensive and evident, leading to kidney hypoperfusion and hepatic ischemia-reperfusion, and even resulting in renal secondary distant pathological damage. Therefore, previous studies have hypothesized that AKI mainly occurs during the perioperative period (2,9). In the present study, ulinastatin was administered during surgery to explore its kidney protective effect. The effect of ulinastatin application on the incidence of AKI and patient prognosis were investigated in clinical trails, while the underlying mechanism of ulinastatin was examined using a rat autologous OLT (AOLT) model.

## Materials and methods

### Patient data and allocation

In total, 60 patients (male, 57; female, 3) with normal renal function and classified as American Society of Anesthesiologists standard grade III-IV, undergoing an OLT at the Third Affiliated Hospital of Sun Yat-sen University (Guangzhou, China), were included in the study. Informed consent was obtained from all the individuals enrolled in the study, and the experimental protocol was approved by the Ethics Committee of the Third Affiliated Hospital of Sun Yat-sen University (no. chiCTR-DNRC-08000085). The median age of the patients was 43.72±8.63 years (age range, 28–68 years), and the Model for End-Stage Liver Disease (MELD) score was calculated for each patient (9). Patients with a diagnosis of hepatorenal syndrome, septic shock, hypovolemic shock, primary renal disease, diabetes, hypertension, heart disease and retransplantation were excluded from the study.

All the patients undergoing an OLT were randomly divided into two groups, the control group (C group; n=30) and ulinastatin treatment group (U group; n=30). Anesthesia was induced by intravenous (i.v.) injection of midazolam (0.05 mg/kg; Jiangsu Nhwa Pharmaceutical Co., Ltd., Xuzhou, China), propofol (1 mg/kg; AstraZeneca, Caponago, Italy), vecuronium bromide (0.1 mg/kg; Xianju Pharmaceutical Co., Ltd., Hangzhou, China) and fentanyl (3 μg/kg; Humanwell Pharmaceutical Co., Ltd, Yichang, China), and maintained with sevoflurane (0.5–3%; Hengrui Pharmaceutical Co., Ltd., Shanghai, China) and intermittent fentanyl and vecuronium bromide (i.v.).

The patients received a modified piggyback liver transplantation with venous reformation, but without the performance of a venovenous bypass. The surgical procedures regarding the first and second hepatic hilum were similar to a classic OLT procedure; however, the most important difference was the surgical management without short hepatic vein disposal. The vena cava (VC) was interrupted when the first hepatic hilum was disconnected from the back of the liver, using a Satinsky clamp. After the VC of the second hepatic hilum was blocked, the liver was removed. Next, the hepatic vein openings on the anterior wall of the inferior VC (IVC) of the patient were connected, forming an open inverted triangular cuff. The posterior wall of the donor IVC was incised in order to form a wide-opened inverted triangular cuff that matched the opening of the recipient IVC. Subsequently, 400–800 ml fresh frozen plasma was used to flush the graft, after which the portal vein was anastomosed and the VC was ligated and reperfused (10).

In the U group patients, ulinastatin (5,000–8,000 U/kg; Techpool Bio-Pharma Co., Ltd., Guangzhou, China) was intravenously pumped for 30 min during the skin incisions. Subsequent administration of ulinastatin was applied 4 h after the beginning of the surgery. The same volume of normal saline was applied to the patients in the control group, in the same manner as the administration of ulinastatin.

Patient demographic data, including the age, gender, baseline serum creatinine (Cr) level and preoperative MELD scores, were recorded prior to surgery. In addition, the surgery duration, ischemia and reperfusion times were measured. Following the OLT, AKI was diagnosed within three days according to the recommendations of the Acute Kidney Injury Network (11). In addition, the AKI incidence rates, serum levels of cystatin C, blood urea nitrogen (BUN) and Cr, 30-day and one-year survival rates, intensive care unit (ICU) time, ventilation time and hemodialysis rates were measured for all the patients.

### Animals

A total of 40 adult male Sprague-Dawley rats (weight, 220–250 g) were purchased from the Experimental Animal Center of Sun Yat-sen University (Guangzhou, China). All the experiments were performed in accordance with the Animal Care and Use Committee of Sun Yat-sen University, and followed the university Guidelines of Animal Use and Protection, adopted from the Guide for the Care and Use of Laboratory Animals (National Institutes of Health, publication no. 80-23, revised in 1996). The rats were divided randomly into three groups, namely the sham-operation rat group (S-R group, n=10), the control rat group (C-R group; n=10) that underwent AOLT and the ulinastatin rat treatment group (U-R group; n=20). The U-R group was divided further into two equal subgroups who received a medium (UM-R; 50,000 U/kg) or high dose (UH-R; 100,000 U/kg) of ulinastatin.

### Animal surgical procedure

Rat AOLT models were established using a previously reported method (12,13). All the rats were intraperitoneally injected with 10% chloral hydrate (0.3 ml/100 g). During the surgery, the rats were allowed to breathe oxygen on an electric heating pad under a warming light. Prior to blocking the VC, ulinastatin (50,000 U/kg or 100,000 U/kg for the medium and high dose groups, respectively) dissolved in heparin (50 U/ml), or saline, was injected into the caudal vein of the U-R and C-R group rats, respectively. After opening the hepatic veins, the remaining ulinastatin dissolved in protamine sulfate (0.5 mg/ml), or saline, was injected into the caudal vein of the respective groups. By contrast, the sham-operation group (S-R group) rats were subjected to an abdominal incision and portal vein dissociation, followed by suturing of the abdominal incision under anesthesia, without blockade of the blood flow.

### Histology and quantification of renal injury

Kidney sections of the rats from the C-R or U-R groups were obtained 8 h following reperfusion and were stained with hematoxylin-eosin (H&E, Keygen Biotech Co., Ltd, Nanjing, China) and periodic acid-Schiff. The samples were analyzed for tubular cell necrosis, tubular dilation and intratubular detachment (magnification, ×200; Eclipse E200; Nikon, Tokyo, Japan), and were evaluated in a blinded manner by a nephrologist. Abnormalities were graded using a semiquantitative score (range, 0–4+) defined as: 0, no abnormalities; 1+, changes affecting <25% of the tubules; 2+, 25–50%; 3+, 50–75%; and 4+, >75% (14).

### Biomarkers of inflammation and oxidative stress

Renal cortexes were collected from all the rats. The levels of tumor necrosis factor-α (TNF-α) and interleukin-6 (IL-6) were measured using an enzyme-linked immunosorbent assay (ELISA) kit (KeyGen Biotech. Co., Ltd.). In addition, the levels of superoxide dismutase (SOD), hydrogen peroxide (H_2_O_2_), malondialdehyde (MDA) and reactive oxygen species (ROS) were determined using the equivalent assay kits, according to the manufacturer’s instructions (KeyGen Biotech. Co., Ltd.).

### Biomarkers of renal injury

Blood samples were collected from the rats and patients. Serum cystatin C levels were measured using an immunonephelometric method (Dade Behring Marburg GmbH,, Marburg, Germany), and a standard calibration formula was used to estimate the glomerular filtration rate from the result. The serum levels of BUN and Cr were measured using an ELISA kit (Rapidbio, Inc., West Hills, CA, USA), according to the manufacturer’s instructions.

### Statistical analysis

Statistical analyses were performed using SPSS 17.0 statistical software (SPSS, Inc., Chicago, IL, USA) and the data are presented as the mean ± standard deviation. Data obtained from the experimental groups were compared using a two-tailed unpaired t-test. Statistical analysis was performed using analysis of variance, followed by the Bonferonni correction to evaluate the differences between pairs. The least significant difference test was used to examine the homogeneity of variance. In addition, the χ^2^ test was used to compare the differences between rates (AKI incidence rate, AKI III cases, 30-day and one-year survival rates and hemodialysis cases). P<0.05 was considered to indicate a statistically significant difference.

## Results

### Patient demographics and outcomes

Table I shows the demographic information and clinical characteristics of the C and U patient groups. Following the OLT, 18 patients met the criteria for AKI in the C group (60.00%), while the number of AKI patients in the U group was 10 (33.3%). Although the incidence of AKI in the U group was not found to be significantly less compared with the C group (P>0.05), the number of patients developing severe AKI (AKI III) (15) was much higher in the C group compared with the U group (P=0.002). Additionally, administration of ulinastatin improved the prognosis of the patients. The 30-day and one-year survival rates showed no statistically significant difference between the two groups; however, the time the patients remained in ICU, the ventilation time and the hemodialysis rates were evidently higher for patients in the C group (P<0.05).

**Figure 1:**
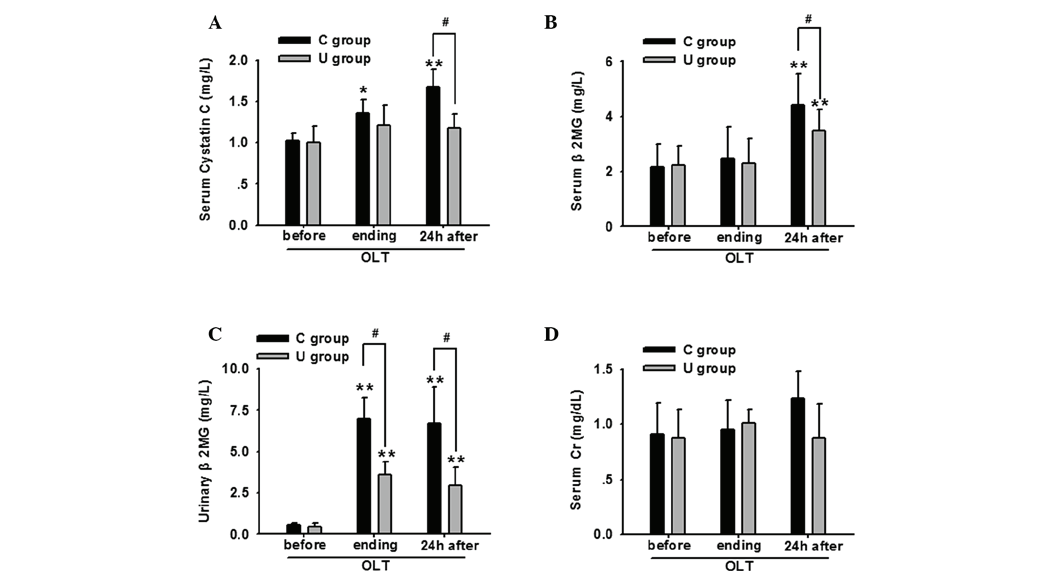
Ulinastatin application significantly decreased the renal damage of OLT patients, as indicated by changes in the serum levels of (A) cystatin C, (B) β_2_MG, (C) urinary β_2_MG and (D) serum Cr at the various time points, including before OLT, the surgery endpoint and 24 h after OLT. ^*^P<0.05 and ^**^P<0.01, vs. before OLT (n=30); ^#^P<0.05, vs. C group at the same time point (n=30). OLT, orthotopic liver transplantation; β_2_MG, β_2_ microglobulin; Cr, creatinine; C group, control group; U group, ulinastatin treatment group.

Notably, the serum cystatin C, β_2_ microglobulin (β_2_MG) and urinary β_2_MG levels increased gradually during the perioperative period, and particularly at 24 h following OLT, indicating that the renal injuries had deteriorated. However, application of ulinastatin decreased the levels of these bioactive substances. Although no statistically significant difference in the serum Cr level was identified between the two groups, a decreasing trend was apparent in the U group (Fig. 1).

### Ulinastatin application significantly decreases the renal damage of rats

**Figure 2:**
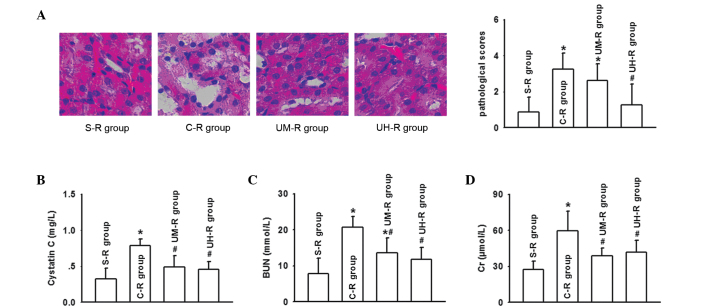
Ulinastatin application significantly decreased the renal damage in rats, as indicated by (A) the pathological damage (magnification, ×200) and kidney scores (hematoxylin and eosin staining), and changes in the serum levels of (B) cystatin C, (C) BUN and (D) Cr, determined 8 h following autologous orthotopic liver transplantation (AOLT). ^*^P<0.05, vs. S-R group; ^#^P<0.05, vs. C-R group. BUN, blood urea nitrogen; Cr, creatinine; S-R group, sham-operation rat group (n=10); C-R group, control rat group (n=10; undergoing AOLT); UM-R group, rats receiving a medium dose of ulinastatin (50,000 U/kg); UH-R group, rats receiving a high dose of ulinastatin (100,000 U/kg).

Histological examination of the kidney was performed 8 h following reperfusion on the rat AOLT models. The results demonstrated that the kidney damage of the rats that had undergone an AOLT was extensive, and multifocal acute tubular injury was observed through the loss of a brush border, the flattening and loss of the tubular epithelium, hyaline casts, medullary congestion and hemorrhage. However, ulinastatin application was shown to improve the tubular injury and decrease the pathological scores effectively, particularly at a high dose (Fig. 2A). Compared with the S-R group, the serum levels of BUN, Cr and cystatin C in the C-R group were significantly increased and peaked at 8 h following reperfusion after AOLT, while ulinastatin application lowered the increase in the serum levels, in accordance with the changes to the kidney pathological injuries (Fig. 2B and D).

### Ulinastatin application decreases renal inflammatory reactions and oxidative stress

**Figure 3:**
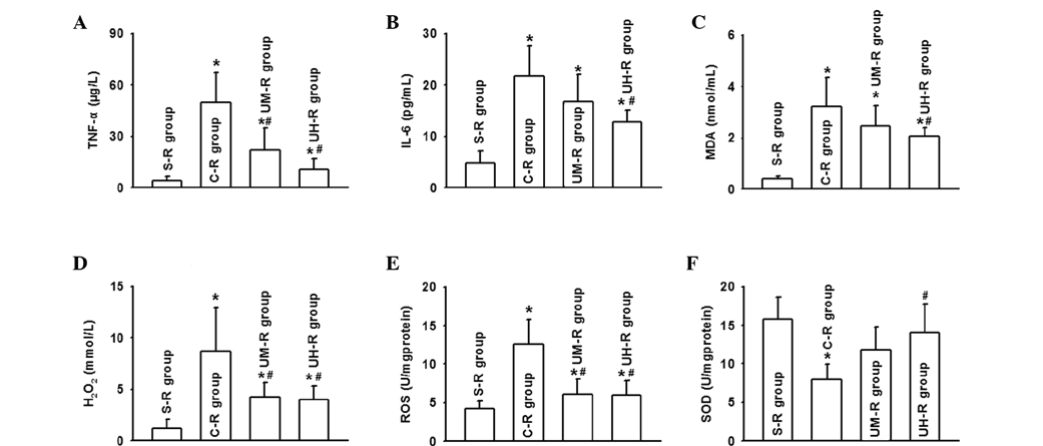
Ulinastatin application attenuated the oxidative stress and inflammatory reaction, particularly in the UH-R group, by reducing the serum levels of (A) TNF-α, (B) IL-6, (C) MDA, (D) H_2_O_2_ and (E) ROS, and (F) increasing the serum level of SOD, determined 8 h following autologous orthotopic liver transplantation (AOLT). ^*^P<0.05, vs. S-R group; ^#^P<0.05, vs. C-R group. TNF, tumor necrosis factor; IL, interleukin; MDA, malondialdehyde; H_2_O_2_, hydrogen peroxide; ROS, reactive oxygen species; SOD, superoxide dismutase; S-R group, sham-operation rat group (n=10); C-R group, control rat group (n=10; undergoing AOLT); UM-R group, rats receiving a medium dose of ulinastatin (50,000 U/kg); UH-R group, rats receiving a high dose of ulinastatin (100,000 U/kg).

As aforementioned, ulinastatin application was shown to protect against renal damage following AOLT; however, the underlying mechanisms have yet to be reported. Severe kidney damage was found to be accompanied with oxidative stress and inflammatory reaction activation. The levels of TNF-α and IL-6 increased markedly, in accordance with changes in the levels of MDA, H_2_O_2_ and ROS during the perioperative period, while the SOD levels decreased. However, the application of ulinastatin effectively balanced the oxidative stress and inflammatory reaction, particularly at a high dose. The levels of the inflammatory factors, TNF-α and IL-6, were decreased, as well as the levels of MDA, H_2_O_2_ and ROS, while the level of SOD increased (Fig. 3). Therefore, the protective function of ulinastatin was considered to be associated with its anti-inflammatory and antioxidant effects.

## Discussion

Renal damage has been demonstrated to be closely associated with the prognosis of patients suffering from OLT. Thus, the American Society of Nephrology has indicated that the diagnosis of renal failure may be replaced by AKI, which may aid the earlier prognosis of renal failure and provide effective prevention for the further deterioration of renal function in patients. AKI is a common and significant complication of liver transplantation, and is responsible for the poor prognosis of OLT patients (16–18). However, no effective methods for the prevention of AKI are currently available. In the present study, the effects of a broad-spectrum protease inhibitor, ulinastatin, were examined. Clinical trial results revealed that ulinastatin application decreased the AKI incidence following OLT, particularly for severe AKI, while improving the prognosis of patients. In a rat AOLT model, ulinastatin application was shown to protect against AKI following surgery and regulate oxidative stress and the inflammatory reaction.

Liver transplantation can result in complicated pathophysiological changes, including hemorrhage, intensive cycle fluctuations induced by vascular occlusion and opening, intestinal congestion and damage and liver ischemia-reperfusion injury. Trauma caused by these changes activates the systemic oxidative stress and inflammatory reaction, leading to multiple organ complications, ultimately affecting the prognosis of patients undergoing OLT (19–21).

A previous study using a rabbit model indicated that ulinastatin application attenuated lung injury by inhibiting the release of inflammatory mediators (22). Yang *et al* further demonstrated that ulinastatin protected liver function and improved the clinical outcomes of patients undergoing a hepatectomy, possibly through the inhibition of inflammation and oxidation at an earlier stage (23). Other studies have also reported that the protective effects of ulinastatin may be associated with the SOD level increase (24) and membrane stabilization in rat models (25). These observations demonstrate that ulinastatin is a promising drug for organ protection. In addition, the clinical trial results of the current study revealed that ulinastatin application was beneficial for patients undergoing OLT. Ulinastatin decreased the incidence of AKI and was particularly effective in severe AKI cases. Although no statistically significant differences were observed between the 30-day and one-year survival rates, ulinastatin application reduced the ICU and ventilation times and hemodialysis rate. Thus, ulinastatin is recommended as a protective strategy used during the perioperative period of OLT to improve patient prognosis.

Oxidative stress and inflammatory reactions induced by the trauma caused by OLT are considered to be the main mechanisms for the formation of AKI resulting from ischemia and toxins (26,27). Ischemia-reperfusion injury, intestinal endotoxemia and the disruption of the internal environmental balance prompt the deterioration of AKI. Therefore, anti-inflammatory and antioxidant processes during the early stages of OLT may be significant in the reduction of AKI incidence (28,29). In the present study, the levels of TNF-α and IL-6 were found to evidently increase in the rat AOLT model, reflecting the degree of the inflammatory reaction (30,31). In addition, the levels of ROS and H_2_O_2_ increased, signifying the activation of oxidative damage, while the increase in the level of MDA indicated the degree of lipid peroxidation (32). These oxidative and inflammatory mediators have been demonstrated to participate in kidney damage induced by various pathogenies, including AKI following AOLT (33,34). In the present study, ulinastatin application was found to be beneficial in the protection against AKI. In order to investigate the possible mechanisms underlying the protective effects of ulinastatin, a rat AOLT model was established and ulinastatin was found to decrease renal pathological damage by inhibiting oxidative stress and inflammatory reactions. These results provide direct evidence of the effect of ulinastatin on kidney protection during OLT.

In summary, ulinastatin was found to attenuate AKI following OLT, partly by inhibiting the inflammatory process and oxidative stress. Therefore, ulinastatin may be a valuable clinical candidate for application during OLT. However, limitations exist in the current study, such as the limited number of clinical samples. Thus, in future studies, an increased sample size should be used and further oxidative and inflammatory mediators should be detected, in order further the understanding of the protective mechanism of ulinastatin. From these studies, a novel strategy for kidney protection in OLT patients may be established.

## Figures and Tables

**Table I:** Comparison of demographic information between the two groups of patients undergoing liver transplantation.

Factor	U group (n=30)	C group (n=30)	P-value
Age, years	42.9±10.7	44.5±8.8	0.521
Female/male, n	1/29	2/28	1.000
Baseline serum creatinine, μmol/l	64.6±16.0	61.9±15.0	0.507
Preoperative MELD score ≥18, n	10	11	0.787
Duration of surgery, min	345.3±66.2	356.8±77.6	0.538
Ischemic time, min	39.7±16.4	37.0±8.6	0.440
Reperfusion time, min	215.5±50	226.3±69.7	0.513
AKI incidence rate, n (%)	10/30 (33.3)	18/30 (60)	0.069
AKI III cases, n (%	) 0/6 (0)	6/6 (100)	0.002
30-day survival rate, n (%)	29/30 (96.7)	26/30 (86.7)	0.353
One-year survival rate, n (%)	27/30 (90)	24/30 (80)	0.472
ICU time, h	56.0±39.4	134.9±105.8	0.000
Ventilation time, h	17.7±8.9	28.2±24.9	0.036
Hemodialysis cases, n (%)	0/4 (0)	4/4 (13.3)	0.029

MELD, Model for End-Stage Liver Disease; AKI, acute kidney injury; ICU, intensive care unit; U group, ulinastatin treatment group; C group, control group.
